# Psychology

**Published:** 1860-10

**Authors:** 


*Psychology.*—Mr. Moreaw has written a work upon Morbid Psychology, in which is proposed the erroneous doctrine, that great geniuses and monomaniacs, alike owe their peculiar condition to a diseased state of the nervous centers. He says: "Genius is owing to an unusual activity of the nervous centers; insanity is also owing to an unusual activity of these centers," his corollary being that they are similar *morbid* conditions. He could as well reason that the function of any organ and its diseases were alike, both depending upon a certain activity. The difficulty is his want of discrimination between a normal and an abnormal activity, a difference as palpable to the scientific man, as is a clear or muddy stream to the ploughboy. B.

